# Supplementary figures and images for: Contribution of ROS and metabolic status to neonatal and adult CD8+ T cell activation
Source: PLoS One. 2019 Dec 16;14(12):e0226388. doi: 10.1371/journal.pone.0226388 (PMC6913967; doi:10.1371/journal.pone.0226388)

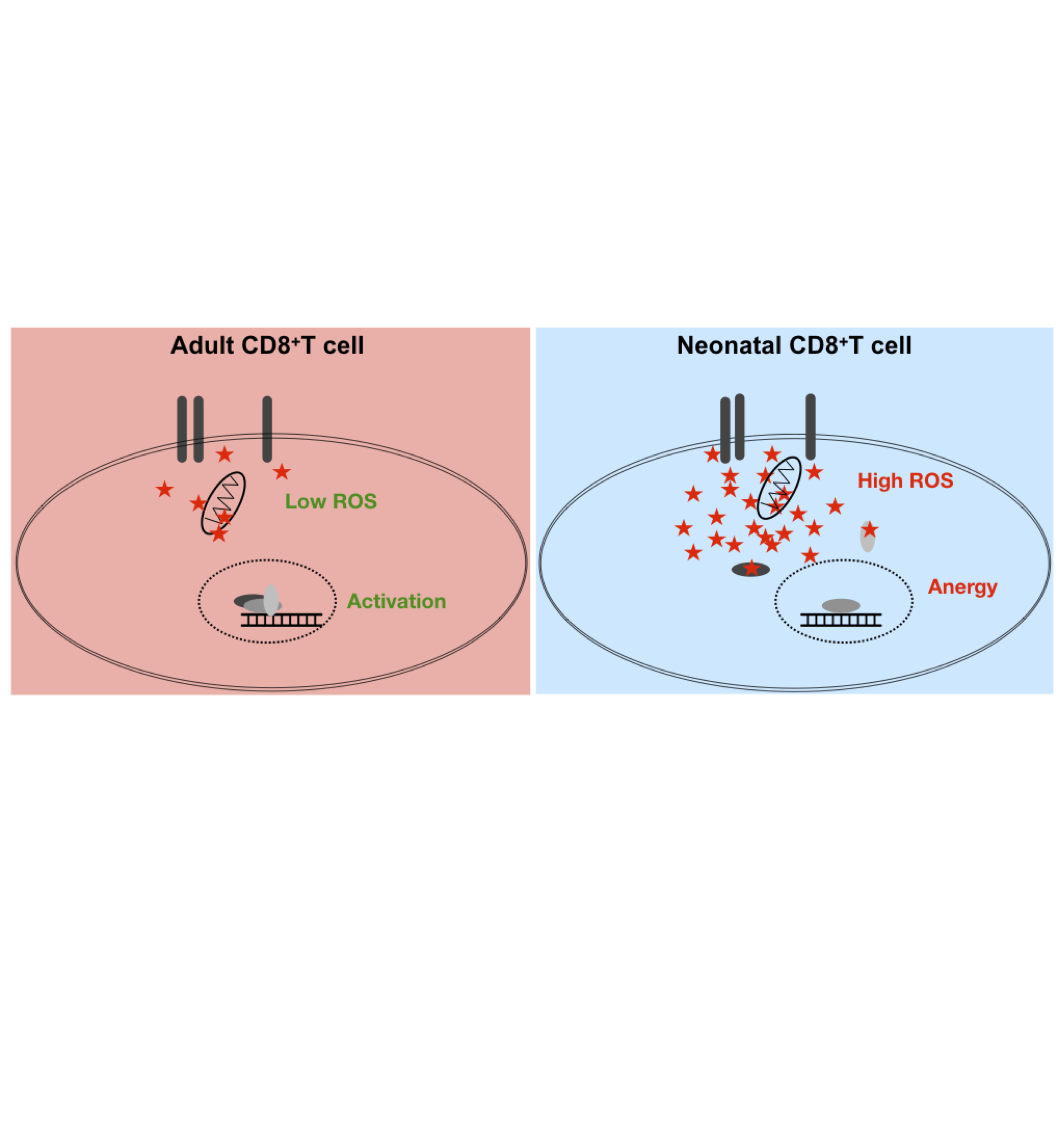

Supplement: S1 Fig — (TIFF) [file pone.0226388.s002.tiff]
